# Female perspective: the burden of Alzheimer's disease and other dementias in China from 1990 to 2019 and prediction of their prevalence up to 2044

**DOI:** 10.3389/fpubh.2023.1101089

**Published:** 2023-04-27

**Authors:** Wenxin Meng, Jinping Xie, Ni Yuan, Pingyu Liu, Fan Yang, Rong Jiang, Hui Hua

**Affiliations:** ^1^School of International Pharmaceutical Business, China Pharmaceutical University, Nanjing, Jiangsu, China; ^2^The Research Center of National Drug Policy and Ecosystem, Nanjing, Jiangsu, China; ^3^Institute of Regulatory Science, China Pharmaceutical University, Nanjing, Jiangsu, China; ^4^School of Public Health, Dalian Medical University, Dalian, Liaoning, China; ^5^The Second Affiliated Hospital of Nanjing Medical University, Nanjing, Jiangsu, China; ^6^School of Health Policy and Management, Nanjing Medical University, Nanjing, Jiangsu, China

**Keywords:** Alzheimer and other dementias, disease burden, China, female, prediction

## Abstract

**Background:**

Dementia is more prevalent in women than in men across the world, and sex differences are reflected in the burden of dementia borne by women and men. However, a few studies have specifically analyzed the disease burden of dementia in Chinese women.

**Objective:**

This article aims to raise awareness of Chinese females with dementia (CFWD), outline an effective response to future trends in China from a female perspective, and provide a reference for the scientific formulation of dementia prevention and treatment policies in China.

**Methods:**

In this article, epidemiological data on dementia in Chinese women were obtained from the Global Burden of Disease Study 2019, and three risk factors, namely, smoking, a high body mass index, and a high fasting plasma glucose, were selected for the analysis. This article also predicted the burden of dementia in Chinese women in the next 25 years.

**Results:**

The prevalence of dementia, mortality, and disability-adjusted life year rates increased with age in CFWD in 2019. All three risk factors provided by the Global Burden of Disease Study 2019 showed positive correlations for the effect of disability-adjusted life years (DALYs) rates on CFWD. Among them, a high body mass index had the greatest effect (8%) and smoking had the smallest effect (6.4%). Over the next 25 years, the number of CFWD and its prevalence are expected to be on the rise, while mortality is expected to remain relatively stable and decline slightly, but deaths from dementia will continue to increase.

**Conclusions:**

The situation arising due to the spread of dementia among Chinese women in the future is going to become a serious issue. To reduce the burden of dementia, the Chinese government should prioritize its prevention and treatment. A multi-dimensional, long-term care system involving families, community, and hospitals should also be established and supported.

## 1. Introduction

Alzheimer's disease is a progressive neurodegenerative disease. It is the most common type of dementia, and the main clinical manifestations of this disease are cognitive impairment, depression, apathy, and unusual repetitive omissions of memory (1, 2). China has a growing population and has been displaying an aging trend; therefore, the prevalence and mortality of Alzheimer's disease and other dementias in China continue to increase, which negatively affects patients, their families, and society. It has been estimated that, in future, China will be the country to be the hardest hit by dementia (3). According to the World Alzheimer Report 2021 (4) (published by Alzheimer's Disease International), dementia is **one** of the seven leading causes of death worldwide. It has been currently estimated that more than 55 million people worldwide have dementia, of whom approximately two-thirds are women, and the risk of developing dementia is higher in women than in men. Therefore, it is important to conduct studies on the burden of dementia and emphasize the need for its timely diagnosis and intervention in women.

Many domestic and international studies have demonstrated sex differences in the burden borne by women and men with dementia. From an economic perspective, Yang and Levey (5) have shown that women spend more money on health insurance, Medicaid costs, and costs of care than men, whether as patients or as dependents of patients. Kasajima et al. (6) have modeled the prevalence and economic burden of dementia in Japan and found that women with lower education, dementia or frailty, or both will bear the highest level of costs in long-term care and health care. From an epidemiological perspective, studies on the burden of dementia in China by Huang et al. (7), Tianhuan and Chuanhua (8), Ren et al. (9, 10), and Zhang et al. (11) have concluded that age-standardized prevalence rates (ASPRs) and age-standardized mortality rates (ASMRs) are higher in Chinese women with dementia (CWWD) than in Chinese men with dementia (CMWD). The Global Burden of Disease (GBD) studies in 2016 and 2019 have concluded that more women than men are affected by dementia globally, and the age-adjusted prevalence continues to be higher in women than men (12, 13). Although most studies have shown that the epidemiological and economic burden of dementia is higher in females than males, these studies analyzed the overall burden of dementia globally, or, in particular, regions or countries. Fewer studies have analyzed the sex differences between women and men with dementia or have comprehensively analyzed the burden of dementia patients in China from a female perspective.

Therefore, this article aimed to raise awareness on CFWD, outline an effective response to future trends in China from a female perspective, and provide a reference for the scientific formulation of dementia prevention and treatment policies in China.

## 2. Methods

### 2.1. Data sources

According to the International Classification of Diseases 10 (ICD-10), the three codes for Alzheimer's disease, vascular dementia, and unspecified dementia are G30, F01, and F03, respectively. The type of dementia mentioned in this article refers to Alzheimer's disease and other dementias.

The primary data for this article were obtained from the GBD 2019 database and sourced from the Institute for Health Metrics and Evaluation at the University of Washington, USA (14). In this article, we used CFWD as the target population and selected “prevalence,” “number of patients,” “mortality,” “deaths,” “DALY rates”, and “DALYs” (disability-adjusted life years) by age group as indicators from the database, and “smoking,” “high BMI” (body mass index), and “high fasting plasma glucose” as risk factors to descriptively statistically analyze the current burden of CFWD. The population data were taken from the United Nations World Population Prospects 2022 Revision (https://population.un.org/wpp/Download/Standard/Population/), which covered the global population from 1950 to 2100, by sex and age. The population data of Chinese women by age group from 1990 to 2044 were selected to predict the prevalence of, the number of patients with, mortality from, and death from dementia in Chinese women in 2044.

### 2.2. Statistical analysis

Dementia is a chronic disease of the nervous system, and it is extremely rare to have the disease under the age of 40 years; therefore, this article selected people with dementia over the age of 40 years for analysis (12). The data on the prevalence of dementia, the number of CFWD, mortality, death, DALY rates, and DALYs were obtained from GBD 2019 in a CSV format. Next, redundant and repetitive data, such as country, cause, and sex, were removed. Descriptive statistical analyses were then performed with the pre-processed data table of dementia burden in China in 1990 and 2019 by age groups of 5 years and sex groups using the ggplot2 package in R visualization. All data obtained from GBD 2019 have a 95% uncertainty interval (UI), which includes the measurement error and uncertainties caused by the bias and modeling.

To show the temporal trend in dementia burden from 1990 to 2019, this article measured the estimated annual percentage change (EAPC) of ASPR, ASMR, and age-standardized DALY rates. The calculation was carried out using the rate fitted by a linear regression model, commonly expressed in logarithm, namely, ln (rate) = α + β^*^ (calendar year) + ε, where α denotes the constant term, β represents the annual change per 100,000 in the rates, and ε refers to the error term (15). EAPC was defined as 100 × (exp (β) – 1); the 95% confidence interval (CI) of EAPC was determined by fitting the model (16). We also conducted a visualization and descriptive statistical analysis of the three risk factors (smoking, high BMI, and high fasting plasma glucose) that affected DALYs in CFWD to further investigate the impact of risk factors on the quality of life of CFWD by age group and temporal trends. Moreover, the prevalence of dementia and mortality were projected for the period 2020–2044 by compiling dementia-related epidemiological data and population data in 5-year increments and using the age–period–cohort analysis by the Nordpred package in R. The Nordpred package has been shown in other studies to be a good predictor of the prevalence of the disease in the future (17). Moreover, the predicted prevalence of dementia and mortality from 2020 to 2044 were calculated for the Chinese female population in the corresponding years to derive the number of patients of dementia and deaths from dementia among Chinese women in the next 25 years. The prevalence rate in 2019 was used as the reference, and its annual decrease or increase of 1% was used as an optimistic or pessimistic reference and compared with the predicted results for analysis. The aforementioned analyses were performed using R (version 4.2.1), R Studio (version 2022.07.1+554), and Excel (version 2016).

## 3. Results

### 3.1. Current dementia burden among Chinese women

Supplementary Table 1 presents the prevalence of dementia, ASPR, deaths, ASMR, DALYs, and age-standardized DALY rates of dementia by sex in China in 1990 and 2019, and the temporal trends from 1990 to 2019. The number of CFWD was high at 8,292,840 (95% UI: 7,000,733, 9,619,121) in 2019. The ASPR of 871.7 per 100,000 (95% UI: 736.7, 1,012.6) was attained in 2019, an increase of 0.29 (95% UI: 0.26, 0.33) from 1990. Dementia is a chronic disease; therefore, the mortality was relatively low at 214,710 (95% UI: 51,100, 559,556) and the ASMR decreased by −0.02 (95% UI: −0.19, 0.23) from 1990 to 24.9 per 100,000 (95% UI: 6, 64.5) in 2019. The DALYs value was 3,753,532 (95% UI: 1,720,507, 8,054,393) and the age-standardized DALYs rate increased insignificantly (0.05; 95% UI: −0.12, 0.26) in 1990–2019 399.9 per 100,000 (95% UI: 184.2, 860.7) in 2019. Overall, all indicators were higher in CFWD than in Chinese men with dementia.

**Figure 1:**
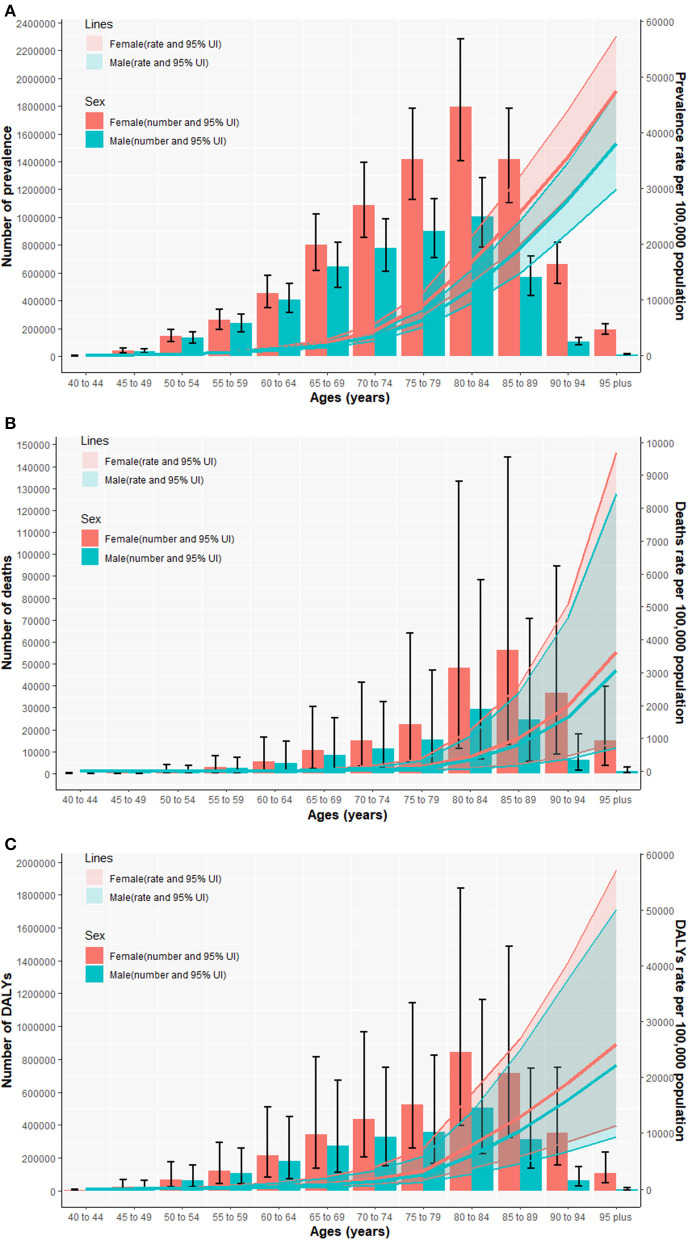
The national numbers and age-standardized rates of prevalence **(A)**, mortality **(B)**, and DALY rates **(C)** of Alzheimer's disease and other dementias per 100,000 population by age and sex in 2019. DALYs, disability-adjusted life years; UI, uncertainty interval.

Further analysis of the burden of CFWD by age groups revealed that the prevalence of dementia, mortality, and DALY rates of Alzheimer's disease that affected female patients increased with age, while the number of CFWD, deaths, and DALYs tended to increase and then decrease with age. The age-standardized prevalence, mortality and DALY rates of CWWD in different age groups are shown in (Figure 1). As shown in Supplementary Tables 2–4, the prevalence of dementia, mortality, and DALY rates in the age group of 60–64 years differed significantly from the values in younger age groups. The prevalence of dementia was below 1,000 per 100,000, mortality below 10 per 100,000, and DALY rates below 500 per 100,000 for female patients under 60 years of age in 2019. By contrast, these values increased significantly for groups over 60 years of age, with the prevalence of dementia up to 47,450.7 per 100,000 (95% UI: 37,974.9, 57,413.1), mortality at 3,631.1 per 100,000 (95% UI: 878.4, 9,686.8), and DALY rates at 25,941.8 per 100,000 (95% UI: 11,296.8, 57,162.7) population in the 95 years plus age group.

Compared with the year 1990, an increase in the prevalence of dementia, mortality, and DALY rates varied by age in the year 2019. The EAPC of prevalence in female patients increased until the age of 70 years and decreased after the age of 74 years, peaking at the ages of 70–74 years (0.8%; 95% CI: 0.67, 0.93), with ~4.7 times the prevalence at the ages of 40–44 years (0.17%; 95% CI: 0.13, 0.22). The mortality EAPC in female patients was negative until the age of 60 years, and then, it fluctuated within a range and peaked in the 95 years plus age group (0.35%; 95% CI: 0.25, 0.45). The EAPCs for DALY rates in female patients up to the age of 59 years increased with age, peaking at the age of 60–69 years (0.39%) and fluctuating within a range thereafter. In terms of sex, the EAPCs for the prevalence of dementia, mortality, and DALY rates were higher for men than for women overall from 1990 to 2019, except for individual age groups in which EAPCs were higher for women than for men.

### 3.2. Risk factors for dementia in Chinese women

**Figure 2:**
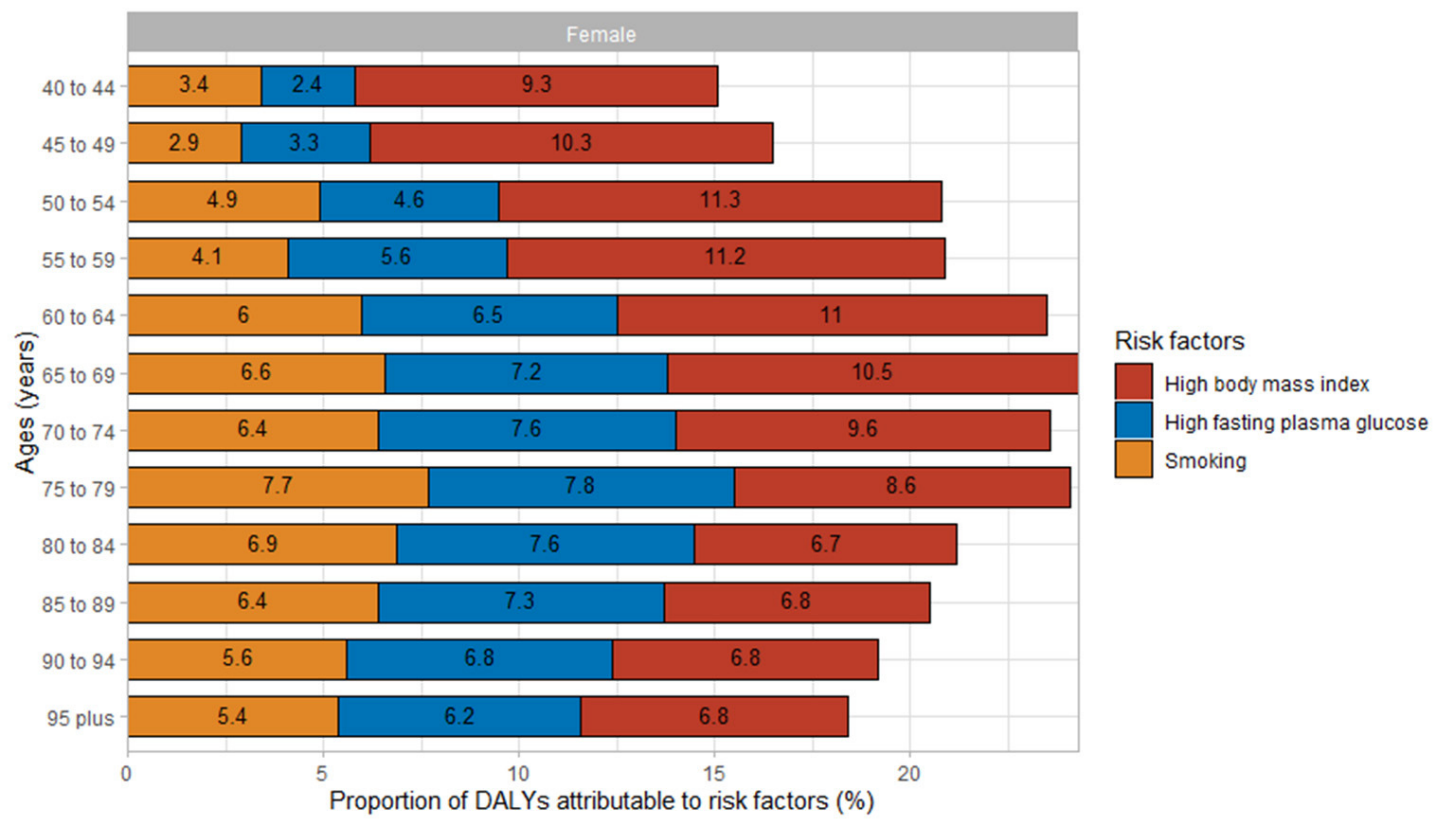
Proportions of DALY rates attributable to risk factors for Alzheimer's disease and other dementias in Chinese women by age in 2019. DALYs, disability-adjusted life years.

The effects of three risk factors on the DALY rates of Chinese women with Alzheimer's disease and other dementias at different ages are shown in Figure 2. Overall, the effects of a high BMI, a high fasting plasma glucose, and smoking on DALY rates showed a positive association; however, the combined effect did not exceed 30%. Among these three risk factors, a high BMI had the greatest effect on DALY rates, with the value of 7.97% (95% UI: 1.56, 18.40). A high fasting plasma glucose was the runner-up, with the value of 7.21% (95% UI: 1.28, 16.98). Smoking had the least effect, with the value of 6.42% (95% UI: 3.51, 9.31). Further analysis of the impact of risk factors on female patients by the age group indicated significant differences in the influence of each risk factor on female patients before the age of 70 years, with a maximum difference of up to 3-fold, while the effect after the age of 74 years was less different, ranging from 0 to 2%. The combined effect of the three risk factors on female patients in each age group tended to increase and then decrease, with the most significant effect observed in the age group of 65–69 years, which was 24.3%.

**Figure 3:**
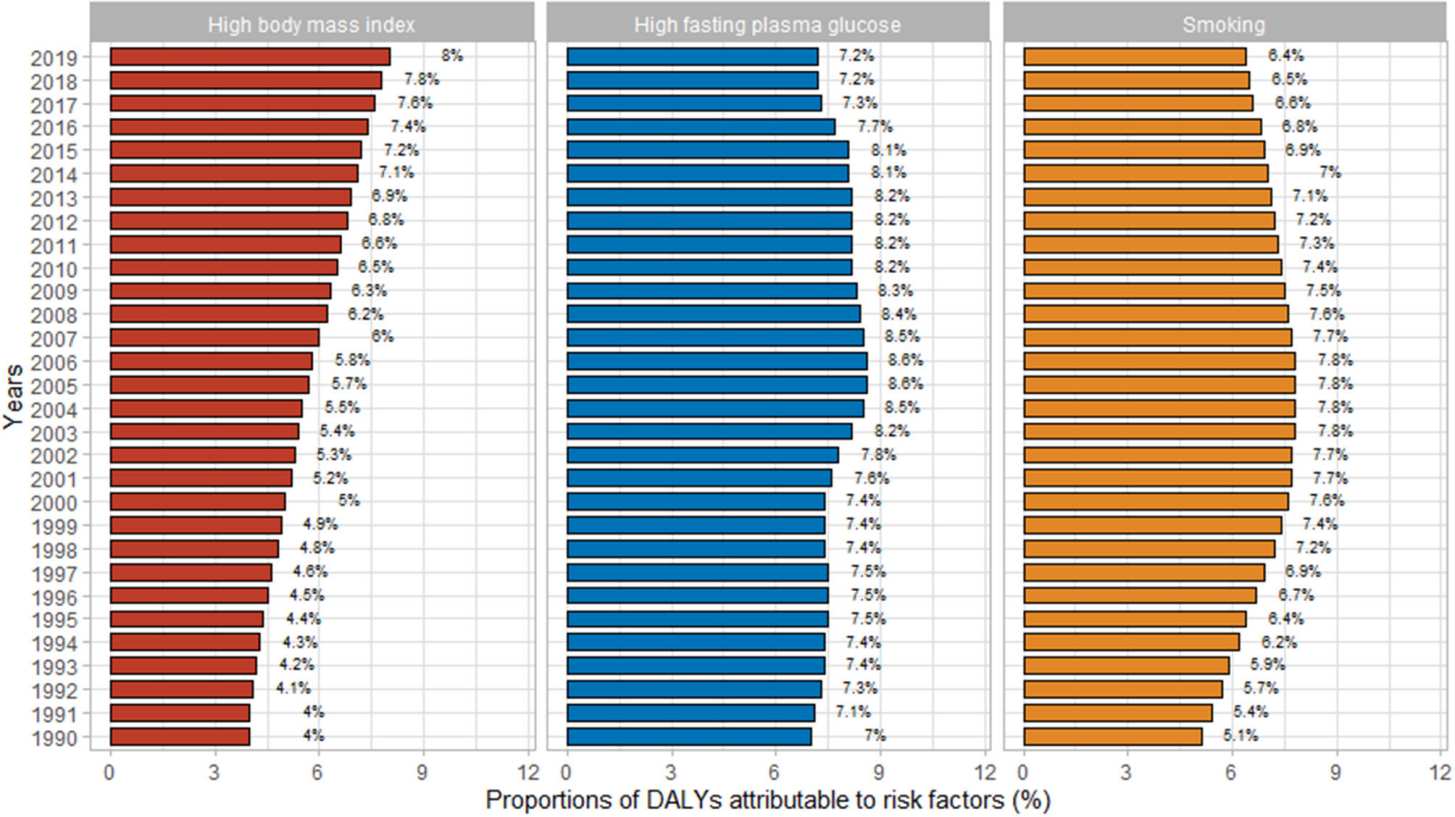
Proportions of Alzheimer's disease and other dementias owing to specific risk factors in Chinese women from 1990 to 2019. DALYs, disability-adjusted life years.

There was a trend in the effect of each risk factor on CFWD over time from 1990 to 2019. The effect of a high BMI increased from 1990 to 2019, whereas the effect of fasting plasma glucose and smoking tended to increase and then decrease from 1990 to 2019. The impact of a high BMI was two times as high in 2019 compared with 1990, whereas the impact of a high fasting plasma glucose and smoking from 1990 to 2019 did not change drastically, with differences ranging from 0.2 to 1.3% (Figure 3).

### 3.3. Trends in dementia among Chinese women

**Figure 4:**
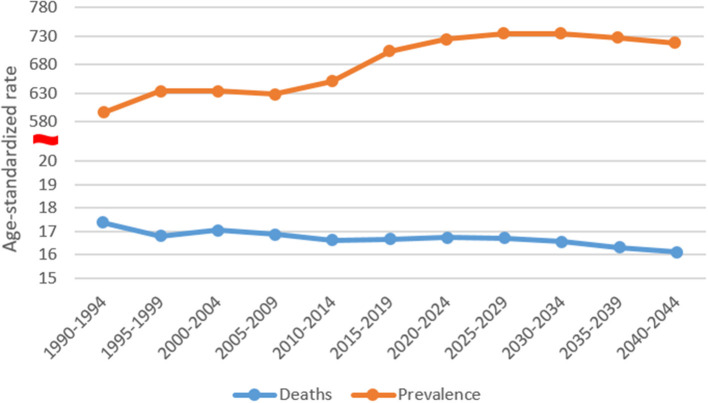
Trends in the prevalence and death rates of Alzheimer's disease and other dementias in Chinese women: observed and predicted rates.

Figure 4 illustrates the trends in the prevalence and death rates of Alzheimer's disease and other dementias in Chinese women from 1990 to 2019 and of their prediction for the period 2020–2044. Owing to the non-fatal nature of dementia, the mortality was low and decreased slightly from 17.37 to 16.1 per 100,000 in 1990-2044, while the prevalence increased by 20.6%, with the most pronounced increase (8.1%) from 2010 to 2019 (Supplementary Table 5).

**Figure 5:**
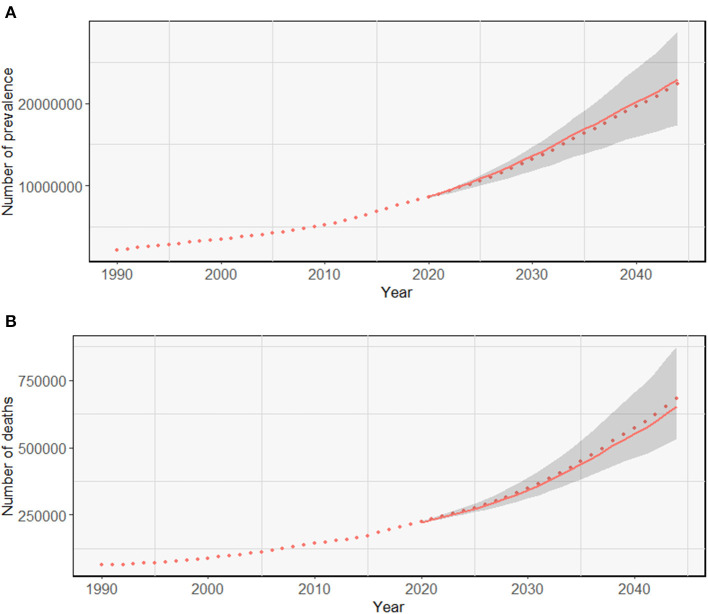
Trends in the observed and predicted prevalence **(A)** and death rates **(B)** of Alzheimer's disease and other dementias. The solid line shows the number of CFWD and deaths in the next 25 years. The dashed line represents the number of CFWD observed and the predicted number of CFWD with the same prevalence and deaths as in 2019. The shaded parts indicate the situation if the prevalence and mortality decrease or increase by 1% per year from those in 2019.

Based on the prevalence and mortality of CFWD in 2019, this article predicted the number of CFWD and deaths in the next 25 years, as shown in Figure 5. It was estimated that 8,292,840 Chinese women had dementia in 2019, increasing to 22,866,775 by 2044, an increase of approximately 176%. The number of deaths was estimated at 214,710 in 2019, increasing to 653,048 by 2044, an increase of approximately 204%. As can be observed from the figure, the predicted number of CFWD is slightly above the reference line, while the number of deaths is slightly below the reference line. Owing to the large female population in China, the projected numbers of CFWD and deaths from dementia are high and the outlook is not optimistic.

## 4. Discussion

Owing to a continuous population growth and an intensifying aging trend, dementia has risen from the 10th to the 5th place in the order of major diseases as causes of death in China from 1990 to 2019 (9, 10). This article demonstrates that the prevalence of dementia, mortality, and DALY rates are high in Chinese women, and the number of CFWD and deaths is expected to increase in the next 25 years. Thus, it is essential to prioritize women's health and plan and allocate healthcare resources appropriately.

In the data from both 1990 and 2019, the prevalence of dementia, mortality, and DALY rates in Chinese women increased with age and the values were consistently higher than those in men. This finding suggests that the burden of dementia is higher in Chinese women than in men, which is consistent with the findings of many previous studies. The decline in the physical function of women as they age, combined with long-term exposure to risk factors, leads to a higher probability of dementia in older women than in middle-aged women. In addition, women usually enter menopause after the age of 40 years, which has a negative effect on metabolism and memory and can reduce protection against cognitive impairment and dementia in terms of vascular health (18). According to the National Bureau of Statistics of China, the average life expectancy of Chinese women exceeds 80 years (19), and the dual status in the family and the workplace imposes increased pressure and burden on women. These factors explain why the prevalence and mortality of CFWD continue to rise, even after the age of 80 years. It also shows the importance of early diagnosis and treatment of dementia. The changes in ASPRs, ASMRs, and age-standardized DALY rates for women from 1990 to 2019 are all lower than those for men, which may be attributed to the rapid economic development, the state's promotion of gender equality, and the increasing social status of women in China during the past 30 years.

In GBD 2019, only three risk factors for dementia were considered: a high BMI, a high fasting plasma glucose, and smoking. Comparing the effect of these three risk factors on women in each age group with the actual prevalence in women, there was a positive correlation between the prevalence and the effect of risk factors in women before the age of 79 years and a negative correlation after the age of 79 years. According to studies by Liu et al. (20), Peters et al. (21), and Choi et al. (22), who analyzed the risk factors affecting dementia, factors such as a lack of physical activity, low educational level, and depression can increase female risk of developing dementia. This conclusion suggests that the prevalence of dementia in women after the age of 79 years is mainly influenced by non-GBD risk factors, such as age, low education, physical inactivity, and depression. The Chinese government has highlighted and supported women's groups in health, education, the economy, social security, and law since 2011; however, there is still a large gap in women's health and social status between urban and rural areas (19). In this regard, the government should target rural women's groups and effectively intervene in risk factor reduction to narrow down the urban–rural gap and reduce the prevalence of CFWD.

Since 1990 to the present, the level of health literacy among Chinese women has steadily increased, as reflected in their dietary habits, lifestyle, and health education. Owing to an increase in the level of education of women, more women are adopting a Western lifestyle, including a high intake of dietary fat, low levels of exercise, smoking, and alcohol consumption. This diet and lifestyle can predispose women to a higher BMI and fasting plasma glucose levels and increase the risk of dementia. Therefore, Chinese women should reduce calorie intake, eat more vegetables and fruits, increase physical activity, and avoid smoking to reduce the risk of dementia. Smoking in China is predominantly prevalent in men; therefore, women are inevitably exposed to tobacco smoke at home, in the workplace, and in public areas. According to the DALYs rate of dementia in Chinese women by risk factors from 1990 to 2019, women should pay attention to their own BMI and fasting plasma glucose levels and avoid smoking.

As the ASPR and the number of CFWD continue to increase over the next 25 years, China should plan ahead, allocate health resources appropriately, and focus on the prevention and treatment of dementia. In traditional Chinese culture and history, women tend to put the needs of their families above their own, thus neglecting their own health needs, which is more common in rural areas. In this regard, scientific education should be strengthened to improve women's knowledge of dementia prevention and treatment, taking into account the characteristics of female patients and high-risk groups. Women should be encouraged to undergo regular cognitive function assessments to improve dementia prevention and treatment. Policies should also be adopted to establish and improve long-term care insurance systems. Furthermore, high-quality medical and health resources should be introduced to rural areas to narrow down the urban–rural gap. In addition, contemporary women need to balance work and family effectively to maintain a healthy lifestyle and reduce stress levels. Women can also experience postpartum depression and negative symptoms of menopause, which can greatly increase the risk of dementia. In this regard, the government and society are called upon to raise the profile of women's group and attach importance to psychological guidance, health needs, and social security. A multi-dimensional model of dementia prevention and intervention for families, communities, and hospitals should be established to improve the capacity of dementia prevention and treatment services.

In terms of resource allocation, a recommendation is to improve long-term care systems based on home care, supplemented by community care and care in medical institutions (23). Currently, home caregivers have insufficient knowledge and skills to deliver professional dementia care to CFWD. Human resources for community and medical institution care are insufficient, the service quality is low, and the gap between urban and rural areas is significant. Therefore, it is advised to provide professional dementia care training for family caregivers in response to the unique pathological mechanisms and risk factors of Chinese women. Especially for CFWD who have lost their spouses in later life, health and community services should be available to support home caregivers to ensure the quality of life of CFWD. Furthermore, memory clinics should be promoted in communities and medical institutions, multidisciplinary care teams should be established, and caregivers regularly trained in dementia care for female patients.

There are some limitations to this article. First, the epidemiological data used were obtained from GBD 2019, which only presents data on the burden of dementia through to 2019. Second, many risk factors, such as low education, lack of physical activity, hypertension in middle-aged adults, diabetes, and others, affect dementia, whereas only three risk factors were covered in GBD 2019. Third, the current situation varies among provinces and cities in China, with large disparities. However, this article only focused on CFWD at the national level.

## 5. Conclusion

This article found that the three assessed risk factors, such as a high BMI, a high fasting plasma glucose, and smoking, were found to be positively associated with the prevalence of dementia in women and that the number of CFWD and deaths will continue to increase in the next 25 years. Thus, the future situation of CFWD is going to be serious and the disease burden of dementia is also going to increase. As there is no effective therapy for dementia, it is advisable to focus on the prevention of dementia in women. The government can enhance women's awareness on the need to prevent dementia by strengthening scientific education and risk factor interventions. Moreover, it could provide policy incentives to improve the long-term care insurance system for chronic diseases to provide a better provision of care. High-quality medical resources should be targeted at rural areas to narrow down the urban–rural gap in women's health. In terms of resource allocation, a multi-dimensional, long-term care system for families, communities, and hospitals should be established. Communities and hospitals need to promote the establishment of memory clinics to provide women with appropriate counseling on dietary habits and lifestyles, as well as on early diagnosis and follow-up services, and train caregivers on a regular basis. Family caregivers should also receive training in caregiving to deliver professional care services to CFWD. This study hopes to protect the rights and interests of the female population and reduce the prevalence and burden of dementia in Chinese women, through the joint efforts of multiple parties.

## Data availability statement

The original contributions presented in the study are included in the article/Supplementary material, further inquiries can be directed to the corresponding authors.

## Author contributions

HH and RJ identified Alzheimer's disease and other dementias as the study topic and Chinese women as the study population and were responsible for manuscript review and revising. PL, NY, and FY were aware of the GBD database and provided relevant data. WM and JX performed data analysis and wrote the article. All authors agreed to the contributions made in this study and reviewed and approved the final manuscript before submission.
