# Supplementary material for: Female perspective: the burden of Alzheimer's disease and other dementias in China from 1990 to 2019 and prediction of their prevalence up to 2044
Source: Front Public Health. 2023 Apr 27;11:1101089. doi: 10.3389/fpubh.2023.1101089 (PMC10173304; doi:10.3389/fpubh.2023.1101089)
Supplement: Supplementary file 1 [file Data_Sheet_1.docx]

Supplementary Material

**Supplementary Table 1.** Prevalence, Death and DALYs of Alzheimer's disease and other dementias in 1990 and 2019 for both sexes and percentage change of age-standardized rates per 100000

|  | | **1990** | | **2019** | | **Percentage change in age-standardized rates between 1990 and 2019** |
| --- | --- | --- | --- | --- | --- | --- |
|  |  | **Counts**  **(95% UI)** | **Rate**  **(95% UI)** | **Counts**  **(95% UI)** | **Rate**  **(95% UI)** |  |
| **Prevalence** | **Male** | 1223516  (1017657,1430125) | 497  (417.3,576.5) | 4851110  (4022835,5654001) | 663.9  (555.7,769.5) | 0.34  (0.30,0.37) |
|  | **Female** | 2122457  (1782794,2472986) | 673.8  (571.6,783.2) | 8292840  (7000733,9619121) | 871.7  (736.7,1012.6) | 0.29  (0.26,0.33) |
| **Death** | **Male** | 30472  (7013,87061) | 19.1  (4.3,53.2) | 106005  (24781,304485) | 20.3  (4.8,57.4) | 0.06  (-0.09,0.28) |
|  | **Female** | 62843  (14311,177076) | 25.4  (6,69.2) | 214710  (51100,559556) | 24.9  (6,64.5) | -0.02  (-0.19,0.23) |
| **DALYs** | **Male** | 677668  (275157,1647186) | 290.1  (117.7,700.2) | 2223508  (980860,5104972) | 321.3  (141.1,744.4) | 0.11  (-0.05,0.29) |
|  | **Female** | 1168325  (480603,2728266) | 381.4  (157.3,876.1) | 3753532  (1720507,8054393) | 399.9  (184.2,860.7) | 0.05  (-0.12,0.26) |

**Supplementary Table 2 (A).** The prevalence cases and age-standardized prevalence of Alzheimer's disease and other dementias in 1990 and 2019 and its temporal trends from 1990 to 2019

| **Male** | **1990** | | **2019** | | **1990-2019** |
| --- | --- | --- | --- | --- | --- |
| **Ages** | **Prevalence cases**  **No. (95% UI)** | **ASPR per 1000000**  **No. (95% UI)** | **Prevalence cases**  **No. (95% UI)** | **ASPR per 1000000**  **No. (95% UI)** | **EAPC in ASPR**  **No. (95% CI)** |
| **40-44** | 2632  (1300,4279) | 7.5  (3.7,12.1) | 3932  (1939,6525) | 7.6  (3.7,12.6) | 0.16  (0.1,0.21) |
| **45-49** | 16257  (10514,23003) | 59.6  (38.5,84.3) | 39022  (25059,55011) | 63.1  (40.5,89) | 0.28  (0.24,0.32) |
| **50-54** | 47384  (33926,63387) | 187.3  (134.1,250.5) | 132447  (94265,175476) | 210.8  (150,279.2) | 0.47  (0.42,0.51) |
| **55-59** | 93314  (70023,118945) | 409.6  (307.4,522.1) | 237370  (175824,302282) | 498.2  (369,634.5) | 0.69  (0.64,0.73) |
| **60-64** | 143315  (110611,182887) | 785.4  (606.2,1002.3) | 409584  (313781,524257) | 1037.9  (795.1,1328.4) | 0.87  (0.81,0.92) |
| **65-69** | 182382  (138575,232674) | 1355.5  (1029.9,1729.2) | 646156  (494553,818101) | 1870.4  (1431.5,2368.1) | 0.91  (0.81,1) |
| **70-74** | 210993  (161764,272739) | 2413.2  (1850.1,3119.4) | 782191  (608986,990198) | 3353  (2610.5,4244.7) | 0.89  (0.78,0.99) |
| **75-79** | 228828  (177683,292067) | 4660  (3618.5,5947.9) | 901754  (707273,1130504) | 6378.8  (5003.1,7996.9) | 0.86  (0.76,0.96) |
| **80-84** | 194115  (149146,251187) | 8922.7  (6855.6,11546) | 1004237  (782816,1285689) | 11990.6  (9346.9,15351.2) | 0.85  (0.77,0.94) |
| **85-89** | 86606  (66035,110614) | 14476.5  (11037.9,18489.4) | 572455  (439704,721386) | 19225.8  (14767.4,24227.6) | 0.85  (0.78,0.93) |
| **90-94** | 15153  (11339,19323) | 21128.2  (15809.6,26942.9) | 108194  (84748,133898) | 28005.7  (21936.6,34659.1) | 0.83  (0.75,0.91) |
| **95 plus** | 2536  (1919,3248) | 29577.2  (22376.4,37887.5) | 13767  (10805,17071) | 38061.3  (29871.8,47196.4) | 0.68  (0.58,0.78) |

**(B)**

| **Female** | **1990** | | **2019** | | **1990-2019** |
| --- | --- | --- | --- | --- | --- |
| **Ages** | **Prevalence cases**  **No. (95% UI)** | **ASPR per 1000000**  **No. (95% UI)** | **Prevalence cases**  **No. (95% UI)** | **ASPR per 1000000**  **No. (95% UI)** | **EAPC in ASPR**  **No. (95% CI)** |
| **40-44** | 2610  (1305,4274) | 8.2  (4.1,13.4) | 4134  (2060,6787) | 8.3  (4.1,13.6) | 0.17  (0.13,0.22) |
| **45-49** | 16140  (10432,22733) | 66.1  (42.7,93.1) | 41811  (26868,58648) | 70.2  (45.1,98.5) | 0.29  (0.25,0.32) |
| **50-54** | 47277  (33865,62734) | 210.2  (150.5,278.9) | 147158  (104238,193725) | 236.4  (167.4,311.1) | 0.45  (0.41,0.49) |
| **55-59** | 95832  (71275,122183) | 463.4  (344.7,590.8) | 262392  (193543,336801) | 556  (410.1,713.6) | 0.62  (0.56,0.67) |
| **60-64** | 156905  (121074,201910) | 913.6  (705,1175.7) | 455540  (348682,580858) | 1165.3  (892,1485.9) | 0.74  (0.68,0.79) |
| **65-69** | 232986  (177610,297928) | 1674.7  (1276.7,2141.5) | 801560  (616941,1021793) | 2236.7  (1721.5,2851.3) | 0.77  (0.68,0.86) |
| **70-74** | 322669  (248852,416123) | 3190.6  (2460.7,4114.7) | 1089425  (853456,1392553) | 4441.6  (3479.5,5677.4) | 0.8  (0.67,0.93) |
| **75-79** | 422238  (331271,540676) | 6495.9  (5096.4,8318) | 1416611  (1125797,1781879) | 9017.4  (7166.2,11342.5) | 0.75  (0.62,0.89) |
| **80-84** | 442891  (343719,568633) | 12787.9  (9924.5,16418.6) | 1796345  (1405506,2282802) | 16800.5  (13145.1,21350.1) | 0.63  (0.52,0.75) |
| **85-89** | 272450  (208677,342509) | 20651  (15817.1,25961.3) | 1420429  (1102945,1780797) | 25699.7  (19955.5,32219.8) | 0.52  (0.43,0.61) |
| **90-94** | 88771  (68589,111264) | 29497.5  (22791.1,36971.6) | 662713  (526081,817110) | 35675.2  (28320,43986.7) | 0.46  (0.37,0.54) |
| **95 plus** | 21687  (16849,27182) | 40414.5  (31397.8,50654.2) | 194722  (155836,235604) | 47450.7  (37974.9,57413.1) | 0.36  (0.27,0.44) |

**Supplementary Table 3 (A).** The number of deaths and age-standardized mortality rates of Alzheimer's disease and other dementias in 1990 and 2019 and its temporal trends from 1990 to 2019

| **Male** | **1990** | | **2019** | | **1990-2019** |
| --- | --- | --- | --- | --- | --- |
| **Age** | **Deaths**  **No. (95% UI)** | **ASMR per 1000000**  **No. (95% UI)** | **Deaths**  **No. (95% UI)** | **ASMR per 1000000**  **No. (95% UI)** | **EAPC in ASMR**  **No. (95% CI)** |
| **40-44** | 41  (4,152) | 0.1  (0,0.4) | 55  (5,192) | 0.1  (0,0.4) | -0.4  (-0.45,-0.35) |
| **45-49** | 222  (29,748) | 0.8  (0.1,2.7) | 440  (54,1441) | 0.7  (0.1,2.3) | -0.43  (-0.5,-0.35) |
| **50-54** | 562  (92,1843) | 2.2  (0.4,7.3) | 1223  (193,3803) | 1.9  (0.3,6.1) | -0.37  (-0.48,-0.26) |
| **55-59** | 1269  (230,3809) | 5.6  (1,16.7) | 2439  (447,7309) | 5.1  (0.9,15.3) | -0.11  (-0.25,0.03) |
| **60-64** | 2227  (470,6346) | 12.2  (2.6,34.8) | 4749  (963,14531) | 12  (2.4,36.8) | 0.19  (0.05,0.33) |
| **65-69** | 3305  (720,9765) | 24.6  (5.4,72.6) | 8431  (1808,25298) | 24.4  (5.2,73.2) | 0.24  (0.11,0.38) |
| **70-74** | 4266  (936,12615) | 48.8  (10.7,144.3) | 11512  (2537,32696) | 49.4  (10.9,140.2) | 0.27  (0.14,0.4) |
| **75-79** | 5267  (1165,16101) | 107.3  (23.7,327.9) | 15590  (3446,47192) | 110.3  (24.4,333.8) | 0.34  (0.2,0.47) |
| **80-84** | 7329  (1643,21607) | 336.9  (75.5,993.2) | 29420  (6551,88274) | 351.3  (78.2,1054) | 0.33  (0.21,0.45) |
| **85-89** | 4669  (1051,13546) | 780.4  (175.7,2264.2) | 24612  (5623,70736) | 826.6  (188.8,2375.7) | 0.37  (0.27,0.47) |
| **90-94** | 1088  (250,3132) | 1517.2  (349.2,4366.8) | 6421  (1459,18019) | 1662.2  (377.5,4664.1) | 0.55  (0.44,0.65) |
| **95 plus** | 227  (51,664) | 2650.6  (592.9,7746.9) | 1112  (257,3054) | 3073.6  (709.8,8444.3) | 0.8  (0.68,0.93) |

**(B)**

| **Female** | **1990** | | **2019** | | **1990-2019** |
| --- | --- | --- | --- | --- | --- |
| **Age** | **Deaths**  **No. (95% UI)** | **ASMR per 1000000**  **No. (95% UI)** | **Deaths**  **No. (95% UI)** | **ASMR per 1000000**  **No. (95% UI)** | **EAPC in ASMR**  **No. (95% CI)** |
| **40-44** | 40  (4,140) | 0.1  (0,0.4) | 52  (5,180) | 0.1  (0,0.4) | -0.65  (-0.7,-0.6) |
| **45-49** | 215  (29,700) | 0.9  (0.1,2.9) | 437  (56,1474) | 0.7  (0.1,2.5) | -0.67  (-0.75,-0.59) |
| **50-54** | 565  (93,1763) | 2.5  (0.4,7.8) | 1320  (203,4178) | 2.1  (0.3,6.7) | -0.5  (-0.59,-0.41) |
| **55-59** | 1323  (242,3716) | 6.4  (1.2,18) | 2787  (502,8201) | 5.9  (1.1,17.4) | -0.01  (-0.17,0.14) |
| **60-64** | 2494  (520,7230) | 14.5  (3,42.1) | 5671  (1175,16549) | 14.5  (3,42.3) | 0.29  (0.15,0.43) |
| **65-69** | 4203  (902,12407) | 30.2  (6.5,89.2) | 10725  (2305,30657) | 29.9  (6.4,85.5) | 0.26  (0.14,0.38) |
| **70-74** | 6337  (1375,17951) | 62.7  (13.6,177.5) | 15149  (3370,41723) | 61.8  (13.7,170.1) | 0.2  (0.1,0.3) |
| **75-79** | 9510  (2082,27556) | 146.3  (32,423.9) | 22306  (5121,64009) | 142  (32.6,407.4) | 0.14  (0.04,0.25) |
| **80-84** | 16325  (3659,47769) | 471.4  (105.6,1379.3) | 48304  (11265,133183) | 451.8  (105.4,1245.6) | 0.01  (-0.08,0.09) |
| **85-89** | 14019  (3276,38760) | 1062.6  (248.3,2937.9) | 56123  (13169,144315) | 1015.4  (238.3,2611.1) | -0.1  (-0.15,-0.04) |
| **90-94** | 5944  (1390,15927) | 1975.3  (461.9,5292.2) | 36935  (8858,94484) | 1988.3  (476.9,5086.2) | 0.12  (0.06,0.18) |
| **95 plus** | 1868  (429,5024) | 3480.5  (799.7,9361.9) | 14901  (3605,39751) | 3631.1  (878.4,9686.8) | 0.35  (0.25,0.45) |

**Supplementary Table 4 (A).** The number of DALYs and age-standardized DALY rates of Alzheimer's disease and other dementias in 1990 and 2019 and its temporal trends from 1990 to 2019

| **Male** | **1990** | | **2019** | | **1990-2019** |
| --- | --- | --- | --- | --- | --- |
| **Age** | **DALYs**  **No. (95% UI)** | **Age-standardized DALY rate per 1000000**  **No. (95% UI)** | **DALYs**  **No. (95% UI)** | **Age-standardized DALY rate per 1000000**  **No. (95% UI)** | **EAPC in age-standardized DALY rates**  **No. (95% CI)** |
| **40-44** | 2289  (504,7493) | 6.5  (1.4,21.2) | 3087  (721,9660) | 5.9  (1.4,18.6) | -0.32  (-0.36,-0.27) |
| **45-49** | 11410  (3138,33660) | 41.8  (11.5,123.3) | 23554  (6964,64529) | 38.1  (11.3,104.4) | -0.28  (-0.35,-0.22) |
| **50-54** | 26757  (8514,73480) | 105.7  (33.6,290.4) | 61927  (21485,155708) | 98.5  (34.2,247.8) | -0.17  (-0.25,-0.08) |
| **55-59** | 52649  (17550,135496) | 231.1  (77,594.7) | 108319  (40001,260360) | 227.4  (84,546.5) | 0.08  (-0.03,0.19) |
| **60-64** | 79423  (29056,197442) | 435.3  (159.2,1082.1) | 181943  (69874,448886) | 461  (177.1,1137.5) | 0.36  (0.25,0.46) |
| **65-69** | 98907  (36633,249412) | 735.1  (272.3,1853.6) | 273747  (109228,672372) | 792.4  (316.2,1946.2) | 0.41  (0.32,0.51) |
| **70-74** | 110612  (45650,272820) | 1265.1  (522.1,3120.3) | 327655  (149816,748658) | 1404.6  (642.2,3209.3) | 0.45  (0.37,0.53) |
| **75-79** | 110777  (47191,271907) | 2255.9  (961,5537.3) | 357491  (166293,824190) | 2528.8  (1176.3,5830.1) | 0.49  (0.41,0.57) |
| **80-84** | 117367  (49184,284339) | 5394.9  (2260.8,13069.9) | 505000  (225545,1160657) | 6029.7  (2693,13858.3) | 0.47  (0.39,0.54) |
| **85-89** | 55773  (22183,134813) | 9322.6  (3708,22534.3) | 310788  (133972,744491) | 10437.8  (4499.4,25003.6) | 0.48  (0.43,0.54) |
| **90-94** | 10073  (3930,24612) | 14045.5  (5479.7,34317.4) | 61985  (26011,144793) | 16044.7  (6732.9,37479.3) | 0.62  (0.55,0.68) |
| **95 plus** | 1630  (657,3973) | 19010.3  (7663.4,46340.9) | 8013  (3357,18131) | 22152.7  (9281.3,50125.2) | 0.68  (0.62,0.75) |

**(B)**

| **Female** | **1990** | | **2019** | | **1990-2019** |
| --- | --- | --- | --- | --- | --- |
| **Age** | **DALYs**  **No. (95% UI)** | **Age-standardized DALY rate per 1000000**  **No. (95% UI)** | **DALYs**  **No. (95% UI)** | **Age-standardized DALY rate per 1000000**  **No. (95% UI)** | **EAPC in age-standardized DALY rates**  **No. (95% CI)** |
| **40-44** | 2202  (494,6796) | 6.9  (1.5,21.2) | 2966  (700,8718) | 6  (1.4,17.5) | -0.51  (-0.55,-0.47) |
| **45-49** | 11064  (3086,31723) | 45.3  (12.6,129.9) | 23704  (7020,66663) | 39.8  (11.8,112) | -0.48  (-0.54,-0.42) |
| **50-54** | 26735  (8469,72893) | 118.8  (37.6,324) | 67006  (23447,174206) | 107.6  (37.7,279.8) | -0.27  (-0.34,-0.21) |
| **55-59** | 54456  (18328,132814) | 263.3  (88.6,642.2) | 122205  (43739,294582) | 258.9  (92.7,624.2) | 0.14  (0.02,0.25) |
| **60-64** | 88102  (31670,223828) | 513  (184.4,1303.3) | 212180  (80752,510261) | 542.8  (206.6,1305.3) | 0.39  (0.3,0.49) |
| **65-69** | 125302  (45778,321918) | 900.7  (329.1,2313.9) | 343740  (136471,813470) | 959.2  (380.8,2269.9) | 0.39  (0.32,0.46) |
| **70-74** | 164518  (69674,389224) | 1626.8  (688.9,3848.7) | 436318  (202729,968590) | 1778.9  (826.5,3948.9) | 0.38  (0.34,0.42) |
| **75-79** | 199644  (86925,468689) | 3071.4  (1337.3,7210.6) | 523138  (257302,1144427) | 3330  (1637.8,7284.8) | 0.33  (0.29,0.37) |
| **80-84** | 260799  (111044,624283) | 7530.3  (3206.3,18025.4) | 844871  (395413,1839349) | 7901.7  (3698.1,17202.7) | 0.18  (0.15,0.22) |
| **85-89** | 167518  (68842,389904) | 12697.4  (5218,29553.7) | 717153  (321112,1487906) | 12975.4  (5809.9,26920.5) | 0.06  (0.03,0.09) |
| **90-94** | 54724  (22517,126148) | 18184  (7482,41917.2) | 353794  (156946,752516) | 19045.5  (8448.7,40509.5) | 0.18  (0.15,0.21) |
| **95 plus** | 13263  (5347,30806) | 24715.7  (9964.7,57406.2) | 106456  (46358,234576) | 25941.8  (11296.8,57162.7) | 0.27  (0.21,0.33) |

**Supplementary Table 5.** Trends in age-standardized prevalence and age-standardized mortality among Chinese women, 1990-2044

| **Year** | **Deaths** | **Prevalence** |
| --- | --- | --- |
| **1990-1994** | 17.37 | 595.52 |
| **1995-1999** | 16.79 | 633.55 |
| **2000-2004** | 17.05 | 633.45 |
| **2005-2009** | 16.86 | 627.69 |
| **2010-2014** | 16.61 | 650.5 |
| **2015-2019** | 16.67 | 703.19 |
| **2020-2024** | 16.72 | 723.85 |
| **2025-2029** | 16.7 | 734.18 |
| **2030-2034** | 16.56 | 734.24 |
| **2035-2039** | 16.32 | 726.82 |
| **2040-2044** | 16.1 | 718.07 |
